# Estrogen Receptor Beta (ERβ) Maintains Mitochondrial Network Regulating Invasiveness in an Obesity-Related Inflammation Condition in Breast Cancer

**DOI:** 10.3390/antiox10091371

**Published:** 2021-08-28

**Authors:** Toni Martinez-Bernabe, Jorge Sastre-Serra, Nicolae Ciobu, Jordi Oliver, Daniel Gabriel Pons, Pilar Roca

**Affiliations:** 1 Grupo Multidisciplinar de Oncología Traslacional, Institut Universitari d’Investigació en Ciències de la Salut (IUNICS), Universitat de les Illes Balears, 07122 Palma de Mallorca, Illes Balears, Spain; toni.martinez@uib.es (T.M.-B.); jorge.sastre@uib.es (J.S.-S.); nicusorciobu@gmail.com (N.C.); jordi.oliver@uib.es (J.O.); pilar.roca@uib.es (P.R.); 2 Instituto de Investigación Sanitaria de las Islas Baleares (IdISBa), Hospital Universitario Son Espases, Edificio S, 07120 Palma de Mallorca, Illes Balears, Spain; 3 Ciber Fisiopatología Obesidad y Nutrición (CB06/03), Instituto Salud Carlos III, 28029 Madrid, Madrid, Spain

**Keywords:** obesity-related inflammation, oxidative stress, mitochondrial biogenesis, mitochondrial dynamics, epithelial-to-mesenchymal transition (EMT), estrogen receptor beta (ERβ), breast cancer

## Abstract

Obesity, a physiological situation where different proinflammatory cytokines and hormones are secreted, is a major risk factor for breast cancer. Mitochondrial functionality exhibits a relevant role in the tumorigenic potential of a cancer cell. In the present study, it has been examined the influence of an obesity-related inflammation ELIT treatment (17β-estradiol, leptin, IL-6, and TNFα), which aims to stimulate the hormonal conditions of a postmenopausal obese woman on the mitochondrial functionality and invasiveness of MCF7 and T47D breast cancer cell lines, which display a different ratio of both estrogen receptor isoforms, ERα and ERβ. The results showed a decrease in mitochondrial functionality, with an increase in oxidative stress and invasiveness and motility, in the MCF7 cell line (high ERα/ERβ ratio) compared to a maintained status in the T47D cell line (low ERα/ERβ ratio) after ELIT treatment. In addition, breast cancer biopsies were analyzed, showing that breast tumors of obese patients present a high positive correlation between IL-6 receptor and ERβ and have an increased expression of cytokines, antioxidant enzymes, and mitochondrial biogenesis and dynamics genes. Altogether, giving special importance to ERβ in the pathology of obese patients with breast cancer is necessary, approaching to personalized medicine.

## 1. Introduction

Cancer, a pathology characterized by the excessive proliferation of tumor cells, is the second cause of death in the world, responsible for approximately 9.6 million deaths in 2018. In addition, breast cancer is a disease causing more than half a million deaths annually, projecting an increase in its incidence to a total of 3.2 million new cases per year in 2050 [1,2]. Breast cancer is a heterogeneous and multifactorial disease that involves both genetic predisposition, lifestyle, and environmental factors. In fact, it has been estimated that about 20% of breast cancer cases around the world are attributed to modifiable risk factors, which include obesity [1].

Obesity is a chronic metabolic disease characterized by excess fat accumulation in the body, and its prevalence has increased markedly in the last two decades in the most developed countries [3]. In obesity conditions, there is an alteration of the adipocyte secretome, which can lead to an imbalance of secreted adipokines, affecting processes as cell proliferation, invasive growth, apoptosis, angiogenesis, and metastasis in tumor cells of tissues such as the mammary gland [4,5,6]. Obesity is a risk factor for breast cancer due to increased circulating estrogens, such as 17β-estradiol. This is explained by the high conversion rate of the androgenic precursors produced by aromatase, an enzyme that increases its activity in the obese state [7].

The cellular effects of estrogens are mediated by their binding to estrogen receptors (ER) alpha (ERα), beta(ERβ), and GPER. Although ERα and ERβ show homology in the DNA and ligand-binding domains, their activity and affinity for 17β-estradiol are different, with ERα having a higher affinity for it. In addition, the expression pattern of the receptors also varies according to the cell type [8,9,10]. ERα is postulated as the main mediator of the carcinogenic effects of 17β-estradiol in breast cancer. In the case of ERβ, which is less studied, it is associated with an antiproliferative, cytostatic, and protective effect against tumor development, although other studies have observed contrary results, so its role in breast cancer remains to be clarified [10,11,12]. Secondly, GPER, also known as GPR30, is a G protein-coupled receptor that can be activated by estrogen to induce effects on proliferation, migration, and invasion, particularly in breast cancer [13].

Another adipokine synthesized and secreted by adipocytes is leptin, whose circulating levels are directly correlated with the individual’s BMI [14]. Leptin has an important role as an independent predictor of risk and prognosis of breast cancer, which has been correlated with its circulating levels. Thus, women with breast cancer have higher plasma leptin levels and RNA expression in adipose tissue than healthy subjects [5,7]. Leptin exerts its biological role by binding to the leptin receptor, which is expressed in normal mammary epithelial cells and breast cancer cell lines, observing an effect on the stimulation of proliferation, cell division, invasion, and metastasis, through the JAK2/STAT3, MAPKs and PI3K/Akt signaling pathways. Furthermore, leptin is also capable of enhancing aromatase expression and activity [11], resulting in increased circulating levels of estrogens, which in turn have been shown to increase mRNA expression and leptin secretion from adipose tissue [14].

It is worth noting that obesity is considered an inducing factor of inflammation, where adipose tissue takes on the role of producer of cytokines and inflammatory proteins. However, the biochemical role between inflammation and cancer-inducing cellular modifications has not yet been elucidated [12]. In this way, the adipose tissue adjacent to the breast tissue is essential in the progression of cancer for the production of proinflammatory cytokines, leptin, and 17β-estradiol [15,16].

The expression of inflammatory cytokines, such as interleukin-6 (IL6) and Tumor necrosis factor alpha (TNFα), have been related to an increase in invasiveness and a poor prognosis in breast cancer [16]. IL6 is secreted by macrophages, fibroblasts, synovial cells, endothelial cells, and keratinocytes, but it is also synthesized by adipocytes [16,17]. Through the interaction with its receptor, IL6 would be able to induce aromatase, inducing a greater synthesis of 17β-estradiol [17]. On the other hand, TNFα is a key inflammatory cytokine produced by macrophages, T cells, B cells, and NK cells, in addition to tumor cells [14]. As a major cytokine in the tumor microenvironment, TNFα influences several of the hallmarks of cancer, such as stimulation of tumor growth, survival, invasion, metastasis, and angiogenesis [18,19]. In breast cancer cell lines, TNFα may have a role in both promoting and inhibiting cell growth, suggesting that the signaling pathway through which it contributes to proliferation is MAPK and PI3K/Akt [5].

Mitochondria is a cellular organelle that is highly influenced in response to estrogens, and it is the main source of reactive oxygen species (ROS), playing an important role in tumor processes, as well as in proliferation and apoptosis [20,21,22]. There is a balance between the production of ROS in the cell and its detoxification by antioxidant enzymes. If antioxidant mechanisms are diminished or ROS production undergoes a significant increase, an imbalance occurs in this system that leads to oxidative damage [23].

Mitochondrial function depends on mitochondria morphology, changes in shape, number, and location, which can translate into important functional modifications. Thus, mitochondrial biogenesis is the combination of both proliferation (an increase in the mitochondrial population) and differentiation (an improvement of the functional capabilities of pre-existing mitochondria) processes [24], while mitochondrial dynamics is a concept that describes the morphology and distribution of mitochondria in the cell [25].

Mitochondrial structure and function can be regulated by the activation of ERα and ERβ receptors by binding of 17β-estradiol [6,26]. Regarding the role of ERβ, some studies have implicated it as a tumor suppressor in breast cancer [6]. ERβ has been shown to colocalize in the mitochondria (mtERβ) and mediate estrogenic effects on the mitochondria, being able to increase mtDNA, increase respiratory capacity, increase antioxidant activity, and inhibit apoptosis [8].

Dysfunctional mitochondria play a relevant role in cancer and in the epithelial-to-mesenchymal transition (EMT) program in breast cancer [27]. As a consequence of mitochondrial defects, deteriorated OXPHOS has been shown to be involved in tumorigenesis [27]. In fact, alteration of mitochondrial functionality has been correlated with a specific mesenchymal phenotype. Moreover, previous studies have hypothesized a link between the regulation of mitochondrial genes, induction of EMT, and metastasis, which implies the worst clinical outcome for patients with cancer [28]. As mentioned above, obesity is an inflammatory disorder in which adipokines, as resistin or leptin, are secreted and play an important role in EMT. It has been described that obesity promotes the metastatic potential of breast cancer by inducing EMT [29]. Moreover, leptin promotes diverse biological events associated with tumorigenesis, as EMT. This process is induced by leptin through the expression of diverse transcription factors, which repress the epithelial markers and promote mesenchymal markers [30].

The aim of the present work was to study mitochondrial functionality and invasiveness, analyzing mitochondrial biogenesis and dynamics processes, as well as oxidative stress and inflammatory status and motility, in cell lines with different estrogen receptors ratio, exposed to a treatment consisting of 17β-estradiol, leptin, IL-6, and TNFα simulating circulating hormonal conditions in a postmenopausal obese woman [31,32,33] Likewise, the expression of the main antioxidant genes and genes related to inflammation and mitochondrial functionality in breast cancer tumors have been studied.

## 2. Materials and Methods

### 2.1. Reagents

Specific reagents 17β-estradiol, leptin, interleukin-6, and TNF-α were purchased from Sigma-Aldrich (St. Louis, MO, USA). Dulbecco’s modified Eagle’s medium without phenol red was purchased from GIBCO (Paisley, UK), Fetal Bovine Serum, and antibiotics solution (penicillin and streptomycin) from Biological Industries (Kibbutz Beit Haemek, Israel). Routine chemicals were supplied by Sigma-Aldrich (St. Louis, MO, USA), Panreac (Barcelona, Spain), and Bio-Rad Laboratories (Hercules, CA, USA).

### 2.2. Cell Culture and Treatments

MCF7 and T47D breast cancer cell lines were purchased from American Type Culture Collection ATCC (Manassas, VA, USA) and maintained in Dulbecco’s modified Eagle’s medium (DMEM) supplemented with 10% Foetal bovine serum (FBS) and 1% antibiotics (penicillin and streptomycin) at 37 °C with 5% CO_2_. To avoid phenol red estrogenic effect, cells were seeded in 6-well or 96-well plates in phenol red-free DMEM containing 10% FBS and 1% antibiotics 24 h prior to treatment. Cells were treated, at 70–80% confluence, with vehicle (0.1% DMSO) or ELIT treatment (10 nM 17β-estradiol, 100 ng/mL leptin, 50 ng/mL interleukin-6 and 10 ng/mL TNFα) for 24 or 48 h.

### 2.3. Measurement of H_2_O_2_ Production and O_2_^−^ Levels

Cells were seeded in 96-well plates (1.6 × 10^4^ cells/well for MCF7 and 3.2 × 10^4^ cells/well for T47D cell line) and treated with ELIT for 48 h. Hydrogen peroxide production was determined by Amplex^®^ Red Hydrogen Peroxide/Peroxidase Assay Kit, following the manufacturer’s protocol, as described previously [34]. Superoxide anion levels were determined by MitoSOX^®^ Red reagent, following the manufacturer’s protocol, as described previously [35]. The values obtained were normalized with the Hoechst 33342 fluorescence signal as previously described [36].

### 2.4. Cardiolipin Content

Cells were seeded in 96-well plates (1.6 × 10^4^ cells/well for MCF7 and 3.2 × 10^4^ cells/well for T47D cell line) and treated with ELIT for 48 h. Cells were stained with 250 nM of Nonyl Acridine Orange (NAO) to measure cardiolipin content as previously described [26]. The values obtained were normalized with the Hoechst 33342 fluorescence signal, as previously described [36].

### 2.5. RT-qPCR

Cells were seeded (4 × 10^5^ cells/well for MCF7 and 6 × 10^5^ cells/well for T47D cell line) in 6-well plates and treated with ELIT for 24 h. Total RNA from cultured cells or breast cancer human biopsies (25 mg) was isolated using TRI Reagent (Sigma-Aldrich, St. Louis, MO, USA), following the manufacturer’s protocol, and then quantified using a BioSpec-nano spectrophotometer (Shimadzu Biotech, Kyoto, Japan) set at 260 nm and 280 nm, getting 260/280 nm ratio. Samples were retrotranscribed to cDNA, and PCR reactions were carried out as previously reported [35]. Genes, primers, and temperatures for the annealing step are specified in Table 1. Both GAPDH and 18S were used as housekeeping genes.

### 2.6. Western Blot

After 48 h of ELIT treatment, cells were harvested as described previously by Torrens-Mas [35]. Protein content (supernatant) was determined with the bicinchoninic acid (BCA) protein assay kit (Thermo Fisher Scientific, Waltham, MA, USA). Ten micrograms of protein were resolved on a 12% SDS-PAGE gel and electrotransferred onto nitrocellulose membranes using the Trans-blot^®^ Turbo™ transfer system (Bio-Rad, Hercules, CA, USA). Membranes were blocked in 5% non-fat powdered milk in TBS with 0.05% Tween for 1 h. Antisera against OXPHOS complexes (ab110411; Abcam, Bristol, UK), SOD-1 (574597, Calbiochem^®^, San Diego, CA, USA), SOD-2 (sc-30080; Santa Cruz Biotechnology, Santa Cruz, CA, USA), CAT (219010, Calbiochem^®^, San Diego, CA, USA), GRd (sc-133245; Santa Cruz Biotechnology, Santa Cruz, CA, USA), 4-HNE (HNE11-S, Alpha Diagnostic, San Antonio, TX, USA), COXIV (ab33985, Abcam, Bristol, UK), PGC1α (ab54481, Abcam, Bristol, UK), and GAPDH (sc-25778; Santa Cruz Biotechnology, Santa Cruz, CA, USA) were used as primary antibodies. Protein bands were visualized as described previously by [34].

### 2.7. Measurement of 4-HNE Adducts Levels

For 4-hydroxy-2-nonenal (4-HNE) adducts analysis, as lipid oxidative damage marker, 20 μg of total protein from cell lysate, processed as previously described by Pons et al. [37].

### 2.8. Wound Healing Assay

Cells were seeded in six-well plates at a density of 1 × 10^6^ cells/well for T47D cell line and 8.5 × 10^5^ cells/well for MCF7 cell line. Wound healing assay was performed as previously described by Torrens-Mas et al. [35]. The area of the scratch was measured using the MRI Wound Healing Tool macro for ImageJ software.

### 2.9. Confocal Microscopy

Cells were seeded on a glass coverslip inside 6-well plates at a density of 2 × 10^5^ cells/well for MCF7 and 5 × 10^5^ cells/well for T47D. After 24 h, cells were treated with ELIT treatment for 24 h. Then, cells were incubated with MitoTracker™ Green 0.5 µM (Invitrogen, M7514) for 1 h and LysoTracker™ Red 0.5 µM (L7528, Invitrogen, Waltham, MA, USA) for 20 min, both at 37 °C in the dark. For DNA staining, cells were incubated with 1/200 dilution of 1 μg/mL Hoechst 33342 (B2261, Sigma, St. Louis, MO, USA) for 5 min at 37 °C in the dark.

The fluorescence was monitored with a Leica TCS-SPE Confocal Microscope, using 63× immersion oil (147 N.A.) objective lens. Fluorescence excitation/emission was 490/516 nm for MitoTracker Green, 577/590 for LysoTracker Red, and 350/455 nm for Hoechst 33342.

Mitochondrial Roundness was analyzed in ImageJ software with Mito-Morphology Macro designed by Ruben K. Dagda at the University of Pittsburgh (2010). This macro is currently maintained and supported by grants NIH/NINDS R01NS105783-01 grant and by NIH/NIGMS R25 1R25-OD023795-01.

### 2.10. Seahorse Metabolic Analyzer

Real-time oxygen consumption rates (OCRs) were determined for MCF7 and T47D cells using the Seahorse Extracellular Flux (XFe96) analyzer (Seahorse Bioscience, North Billerica, MA, USA). Cells were seeded at a density of 4.8 × 10^3^ cells/well for MCF7 and 9.600 cells/well for T47D into XFe96 well cell culture plates and incubated overnight to allow attachment at 37 °C in 5% CO_2_. After 24 h, cells were incubated with vehicle or ELIT treatment. After 48 h of incubation, cells were maintained in 200 μL/well of XF assay media at 37 °C, in a non-CO_2_ incubator for 1 h. During the incubation time, mitochondrial complex inhibitors (1 μM oligomycin, 2 μM FCCP, 0.5 μM rotenone, and 0.5 μM antimycin A) were preloaded for OCR measurements, in XF assay media into the injection ports in the XFe96 sensor cartridge.

### 2.11. Human Samples

Human breast cancer biopsies were obtained from 33 women, ages between 45–90 years. Samples of these patients were obtained from the Biological Specimen Bank of Son Llàtzer Hospital and as specified by and with the necessary permission granted from the Balearic Island Bioethics Committee. Tumor samples were collected immediately after tumor removal and were frozen in isopentane for analysis as described by Sastre-Serra et al. [38]. Written informed consent was obtained from the patients before surgery. All the patients presented an invasive ductal carcinoma (ER-positive, PR-negative, and HER2-negative, as determined by immunohistochemistry) and were classified in normal weight (nw), overweight (ow), and obese (o) by BMI (kg/m^2^).

### 2.12. Statistical Analysis

The statistical analyses were performed with the Statistical Programme for the Social Sciences software for Windows (SPSS, version 27.0; SPSS Inc, Chicago, IL, USA). Data are presented as mean ± standard error of the mean (SEM). The statistical differences in cell lines between vehicle- and ELIT-treated cells were analyzed using a Student’s *t*-test with statistical significance was set at *p* < 0.05 (*). The statistical differences in human samples were analyzed using Pearson’s correlation with statistical significance was set at *p* < 0.01 (**) and *p* < 0.05 (*).

## 3. Results

### 3.1. Obesity-Related Inflammation Treatment Increases Inflammation-Related Genes Expression in Breast Cancer Cell Lines

**Figure 1:**
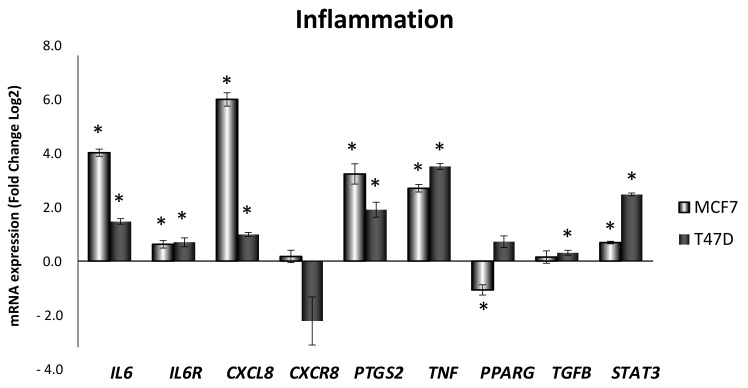
Obesity-related inflammation treatment increased the expression of genes related to inflammation in MCF7 and T47D breast cancer cell lines. *IL6*: interleukin-6; *IL6R*: interleukin-6 receptor; *CXCL8*: interleukin-8; *CXCR8*: interleukin-8 receptor; *PTGS2*: cyclooxygenase-2; *TNF*, tumor necrosis factor alpha; *PPARG*: peroxisome proliferator-activated receptor gamma; *TGFB*: transforming growth factor beta; *STAT3*: Signal transducer and activator of transcription *3*. Breast cancer cells were incubated for 24 h with vehicle (DMSO) or ELIT. Data are represented as fold change (log2) of mRNA expression with respect to vehicle-treated cells, set at 0, of each cell line. Data represent means ± SEM (*n* = 6). * Statistically significant difference between ELIT treated and vehicle-treated cells (Student’s *t*-test, *p* < 0.05).

To confirm the obesity-related inflammation treatment effectivity, mRNA expression of main inflammatory genes was determined. As shown in Figure 1, inflammatory genes expression (*IL6*, *IL6R*, *CXCL8*, *PTGS2*, *TNF*, and *STAT3*) showed a statistically significant increase in both MCF7 and T47D cell lines after 24 h of ELIT (17β-estradiol (10 nM), leptin (100 ng/mL), interleukin-6 (50 ng/mL), and TNFα (10 ng/mL)) treatment. In contrast, *PPARG* anti-inflammatory gene had decreased expression in the MCF7 cell line, and *TGFB* anti-inflammatory gene showed a statistically significant increase in the T47D cell line.

### 3.2. Obesity-Related Inflammation Treatment Increases Oxidative Stress in Breast Cancer Cell Lines with High ERα/ERβ Ratio

As shown in Table 2, ELIT treatment, an increase ROS production in both MCF7 and T47D cell lines was seen. Superoxide anion levels increased more than 10 times in MCF7 cells (1177%) and 5 times in T47D cells (526%) after ELIT treatment, as shown in Table 2, and H_2_O_2_ production increased in both cell lines (+100% in MCF7 and +34% in T47D cells). However, cardiolipin content, as an indicator of mitochondrial inner membrane quantity, decreased in MCF7 treated cells. Moreover, as shown in Table 2, oxidative damage increased (+45%) in MCF7 cell line after ELIT treatment, but not in the T47D cell line. A representative blot of 4-HNE detection is shown in Supplementary Materials Figure S2.

**Figure 2:**
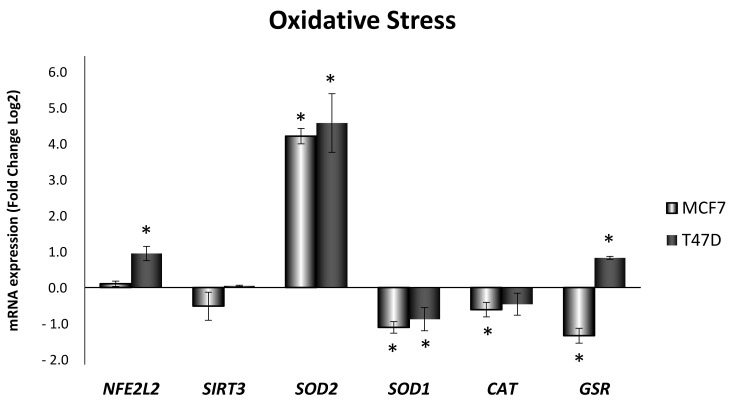
Obesity-related inflammation treatment modified expression of genes related to oxidative stress in MCF7 and T47D breast cancer cell lines. *NFE2L2*: nuclear factor erythroid 2-related factor 2; *SIRT3*: sirtuin 3; *SOD2*: manganese superoxide dismutase; *SOD1*: copper/zinc superoxide dismutase; *CAT*: catalase; *GSR*: glutathione reductase. Breast cancer cells were incubated for 24 h with vehicle (DMSO) or ELIT. Data are represented as fold change (log2) of mRNA expression with respect to vehicle-treated cells, set at 0, of each cell line. Data represent means ± SEM (*n* = 6). * Statistically significant difference between treated and vehicle-treated cells (Student’s *t*-test, *p* < 0.05).

The mRNA expression of antioxidant enzymes was analyzed (Figure 2) in both MCF7 and T47D cell lines after 24 h ELIT treatment. As shown in Figure 2, *SOD2* (mitochondrial superoxide dismutase) showed a high increase in both cell lines; in addition, a decrease in *SOD1* (copper/zinc superoxide dismutase) was observed. However, ELIT-treated MCF7 cells showed a general decrease in catalase (*CAT*) and glutathione reductase (*GSR*). Nevertheless, in the T47D cell line, ELIT treatment increased *GSR* and nuclear factor erythroid 2-related factor 2 (*NFE2L2*) expression, as shown in Figure 2.

Main antioxidant protein expression levels were determined (Table 3 and Supplementary Materials) after 48 h ELIT treatment and resulted in a high increase in SOD2 in both MCF7 and T47D cell lines (more than 10 times in MCF7 and 30 times in T47D). Moreover, SOD1 showed a statistically significant increase in the T47D cell line after treatment. As shown in Table 3, CAT and GSR protein levels decrease only in the MCF7 cell line after ELIT treatment (−30% and −51%, respectively). Representative detection bands are shown in Supplementary Materials Figures S3–S6.

### 3.3. Mitochondrial Biogenesis and Functionality Are Reduced in Breast Cancer Cell Lines after ELIT Treatment with a High ERα/ERβ Ratio

**Figure 3:**
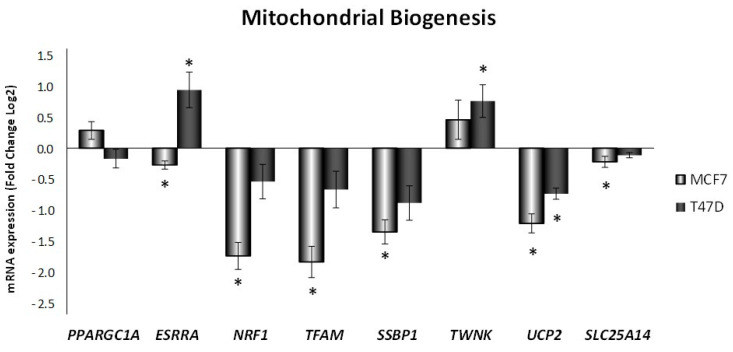
Obesity-related inflammation treatment modified expression of genes related to mitochondrial biogenesis in MCF7 and T47D breast cancer cell lines. *PPARGC1A*: peroxisome proliferator-activated receptor-gamma coactivator-1alpha; *ESRRA*: estrogen-related receptor alpha; *NRF1*: nuclear respiratory factor 1; *TFAM*: mitochondrial transcription factor A; *SSBP1*: mitochondrial single-strand DNA binding protein; *TWNK*: Twinkle mtDNA helicase; *UCP2*: uncoupling protein 2; *SLC25A14*: uncoupling protein 5. Breast cancer cells were incubated for 24 h with vehicle (DMSO) or ELIT. Data are represented as fold change (log2) of mRNA expression with respect to vehicle-treated cells, set at 0, of each cell line. Data represent means ± SEM (*n* = 6). * Statistically significant difference between treated and vehicle-treated cells (Student’s *t*-test, *p* < 0.05).

Mitochondrial biogenesis genes were also checked, as shown in Figure 3. ELIT treatment decreased the expression of almost all mitochondrial biogenesis genes analyzed in MCF7 cell line. Nevertheless, in the T47D cell line, not only did the mRNA expression not decrease but also, especially high levels of estrogen-related receptor alpha (*ESRRA*) and Twinkle (*TWNK*) were found in ELIT-treated cells versus non-treated cells. Moreover, uncoupling proteins 2 and 5 (*UCP2* and *SLC25A14*, respectively) mRNA expression was analyzed, and a decrease in UCP2 expression in both cell lines was accompanied by a decrease in uncoupling protein 5 expression in the MCF7 cell line.

Mitochondrial biogenesis master regulator PPARGC1A protein levels increased after ELIT treatment in the MCF7 cell line. However, to check the effect of ELIT treatment on the mitochondrial respiratory chain (OXPHOS), protein levels of these OXPHOS complexes were analyzed. As seen in Table 4 and Supplementary Materials, ELIT treatment did not change protein levels in T47D, whereas, in MCF7 cell line OXPHOS complexes, protein levels decreased, except complex III (Q-cytochrome c oxidoreductase) after 48 h treatment. Representative detection bands are shown in Supplementary Materials in Figures S7 and S8.

**Figure 4:**
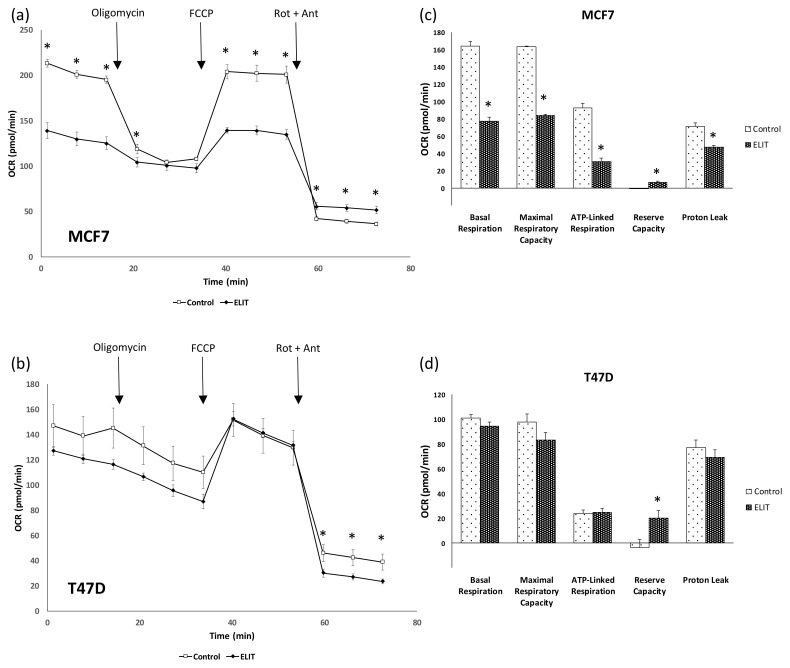
ELIT treatment reduced oxygen consumption ratio (OCR) in the MCF7 cell line. (**a**,**b**): OCR basal conditions and after oligomycin, FCCP, and antimycin A + rotenone addition. (**c**,**d**): calculated parameters from oxygen consumption analysis. Basal respiration: initial rate—antimycin A + rotenone rate; ATP-Linked Respiration: maximal respiratory capacity: FCCP rate—antimycin A+ rotenone rate; initial rate—oligomycin rate; Reserve capacity: FCCP rate—initial rate; proton leak: oligomycin rate—antimycin A+ rotenone rate. Values are expressed as means ± SEM (*n* = 5). * Statistically significant difference between treated and vehicle-treated cells (Student’s *t*-test, *p* < 0.05).

To further investigate mitochondrial function, the oxygen consumption rate (OCR) was determined. As shown in Figure 4a,c, basal OCR, Maximal respiratory capacity, ATP-linked respiration, and proton leak were statistically significantly lower in the MCF7 cell line after ELIT 48 h treatment. These changes were not observed in the T47D cell line (Figure 4b,d). Moreover, reserve capacity, calculated as basal minus maximal respiratory capacity rates, showing an increase in both cell lines, though this increase was higher in the T47D than in the MCF7 cell line.

### 3.4. Obesity-Related Inflammation Treatment Affects Mitochondrial Dynamics and Mitochondrial Network in Breast Cancer Cell Lines

**Figure 5:**
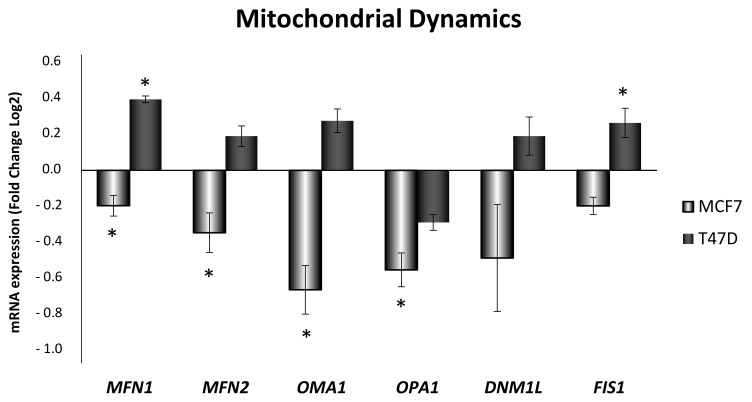
Obesity-related inflammation treatment modified expression of genes related to mitochondrial dynamics in MCF7 and T47D breast cancer cell lines. *MFN1*: mitofusin-1; *MFN2*: mitofusin-2; *OPA1*: mitochondrial dynamin-like GTPase; *OMA1*: zinc metallopeptidase; *DNM1L*: dynamin 1 like; *FIS1*: mitochondrial fission 1 protein. Breast cancer cells were incubated for 24 h with vehicle (DMSO) or ELIT. Data are represented as fold change (log2) of mRNA expression with respect to vehicle-treated cells, set at 0, of each cell line. Data represent means ± SEM (*n* = 6). * Statistically significant difference between treated and vehicle-treated cells (Student’s *t*-test, * *p* < 0.05).

As shown in Figure 5, mitochondrial dynamics genes expression was decreased in MCF7 cells after 24 h ELIT treatment, but in T47D treated cells, this situation was not observed. In fact, almost all the genes showed an increase after treatment in this cell line. The expression of mitochondrial fusion-related genes *MFN1*, *MFN2*, *OMA1*, *OPA1* showed decreased levels in the MCF7 cell line, whereas, in the T47D cell line, ELIT treatment increased expression of *MFN1*, *MFN2*, and *OMA1*. Fission-related mitochondrial genes presented a different pattern expression between cell lines, with a statistically significant increase in *FIS1* mRNA expression in the T47D cell line after ELIT treatment.

**Figure 6:**
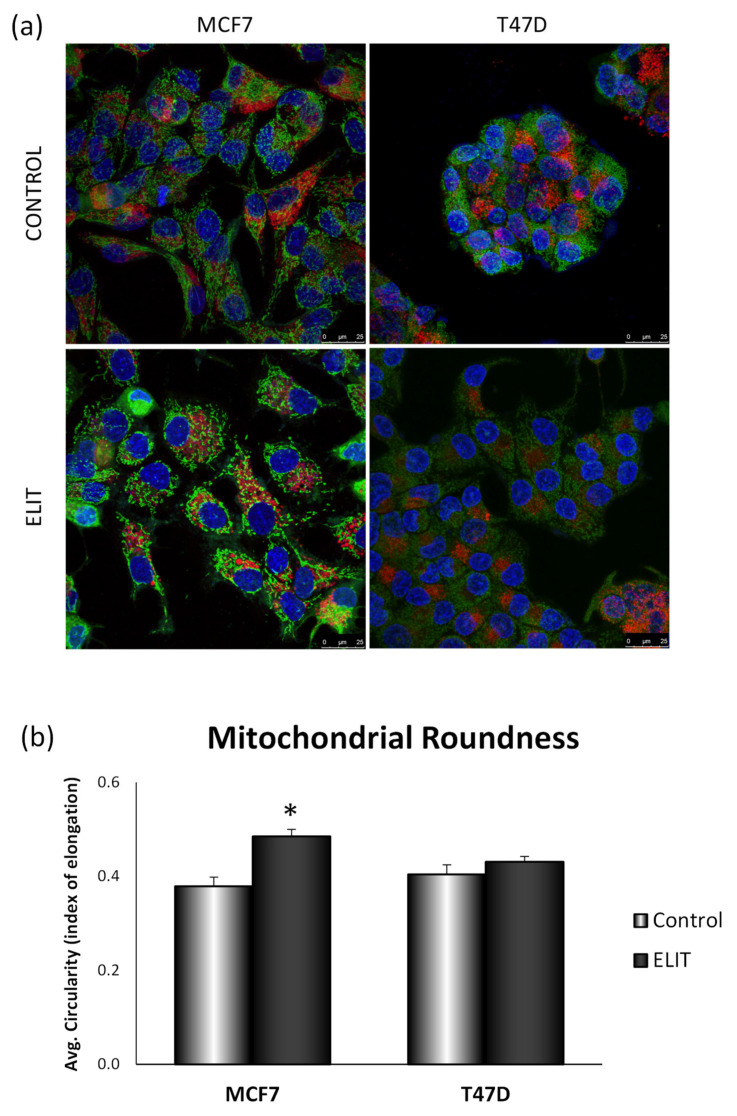
ELIT treatment induces mitochondrial networking changes in the MCF7 cell line. (**a**) Breast cancer cell lines MCF7 and T47D were treated with ELIT for 24 h followed by co-incubation with MTG, LTR, and Hoechst 33342. The fluorescence was monitored with a Leica Confocal Microscope using 63× oil 147 N.A. objective lens. Scale bar 25 µm. (**b**) Mitochondrial Roundness was analyzed with ImageJ Software. Breast cancer cells were incubated for 24 h with vehicle (DMSO) or ELIT. Data are represented as an index of elongation, set at one when a perfect circle. Data represent means ± SEM (*n* = 6). * Statistically significant difference between treated and vehicle-treated cells (Student’s *t*-test, * *p* < 0.05).

On the one hand, mitochondrial networking was modified after 24 h-ELIT treatment in both MCF7 and T47D cell lines. However, as shown in Figure 6a, the MTG signal was higher in the MCF7 cell line after treatment, but LTR intensity was also high. These changes were not observed in T47D cell lines after ELIT treatment. On the other hand, lysosomes distribution in MCF7 cell lines, whereas more equally distributed dispersed through the cytoplasm after 24 h-ELIT treatment, whereas in T47D cell line this distribution showed seemed to be more perinuclear. Index of elongation, calculated as the average circularity of mitochondria of confocal microscopy images, as shown in Figure 6b. As seen, the MCF7 cell line after 24 h ELIT treatment showed more circular mitochondria than in control cells. These morphological modifications were not seen in the T47D cell line with treatment.

### 3.5. Obesity-Related Inflammation Treatment Increase Invasiveness in Breast Cancer Cell Lines with a High ERα/ERβ Ratio

**Figure 7:**
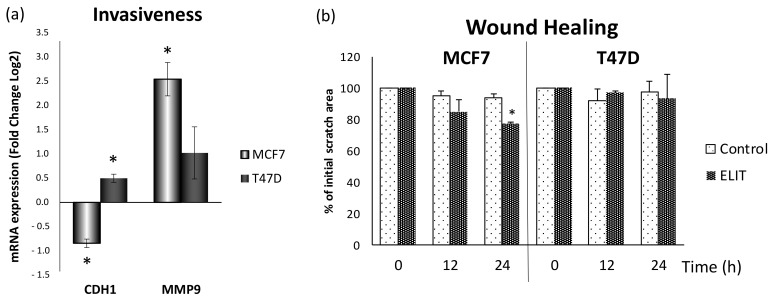
Invasiveness and cell motility were increased after ELIT treatment in the MCF7 cell line. (**a**) CDH1: cadherin-E; MMP9: Matrix Metalloproteinase 9. Breast cancer cells were incubated for 24 h with vehicle (DMSO) or ELIT. Data are represented as fold change (log2) of mRNA expression with respect to vehicle-treated cells, set at 0, of each cell line. Data represent means ± SEM (*n* = 6). (**b**) Area of the wound that remained open after 24 h with ELIT treatment. Data are represented as a percentage of the initial scratch area with respect to vehicle-treated cells of each well, set at 100, of each cell line. Data represent means ± SEM (*n* = 3). * Statistically significant difference between treated and vehicle-treated cells (Student’s *t*-test, *p* < 0.05).

As shown in Figure 7a, Cadherin E (*CDH1*) expression was decreased in MCF7 cells after 24 h ELIT treatment, but Matrix Metalloproteinase 9 (*MMP9*) expression increased three times with respect to vehicle-treated cells. In contrast, CDH1 expression increased in the T47D cell line after treatment. In addition to these results, a wound-healing assay was performed. As shown in Figure 7b and in Supplementary Materials Figure S9, after 24 h, ELIT-treated MCF7 cells were able to better close the wound, leaving 80% of the initial scratch open, while the control cells were not able to close the wound. In the T47D cell line, there were no differences between control and ELIT-treated cells.

### 3.6. Estrogen Receptor Ratio Is Modified by ELIT Treatment in Breast Cancer Cell Lines

**Figure 8:**
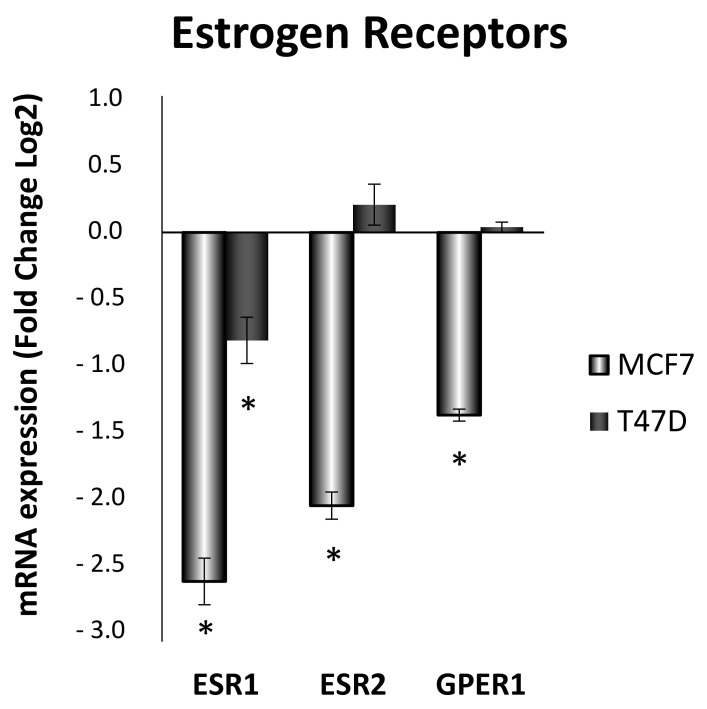
Obesity-related inflammation treatment modified expression of estrogen receptor alpha, beta, and GPER in MCF7 and T47D breast cancer cell lines. *ESR1*: estrogen receptor alpha; *ESR2*: estrogen receptor beta; GPER1: G-coupled protein estrogen receptor. Breast cancer cells were incubated for 24 h with vehicle (DMSO) or ELIT. Data are represented as fold change (log2) of mRNA expression with respect to vehicle-treated cells, set at 0, of each cell line. Data represent means ± SEM (*n* = 6). * Statistically significant difference between treated and vehicle-treated cells (Student’s *t*-test, * *p* < 0.05).

To observe the effects of obesity-related inflammation treatment over estrogen receptors alpha (ERα), beta (ERβ), and GPER in MCF7 and T47D breast cancer cell lines, mRNA expression was analyzed. As shown in Figure 8, estrogen receptor alpha (*ESR1*) mRNA expression was decreased in both cell lines after 24 h-ELIT treatment. However, estrogen receptor beta (*ESR2*) and GPER (*GPER1*) mRNA expression only shown a decrease in the MCF7 cell line, maintaining its expression in the T47D cell line.

### 3.7. IL6R in Breast Tumors Correlates with Inflammation, Mitochondrial Biogenesis, and Oxidative Stress Markers in Different BMI Situations

Table 5 shows the correlation with Pearson correlation values between the IL6R mRNA expression and studied genes in different BMI situations: normal weight (nw), overweight (ow), and obese (o). *CXCR8*, *CXCL8*, *TNF*, *SLC25A14*, *NRF1*, *NFE2L2*, *PPARGC1A*, and *FIS1* were significantly and positively correlated with IL6R expression in the nw group. *CXCR8*, *TNF*, *SLC25A14*, *NRF1*, *NFE2L2*, and *PPARGC1A* were significantly and positively correlated with IL6R expression in the ow group. *CXCR8*, *CXCL8*, *TNF*, *PTGS2*, *ESR2*, *GPX1*, *SOD1*, *SLC25A14*, *NRF1*, *NFE2L2*, *PPARGC1A*, *SIRT1*, *FIS1*, and *OMA1* were significantly and positively correlated with IL6R expression in the ow group.

Moreover, *ESR1* and *ESR2* gene expression correlations are shown in Table 6a,b, respectively. On the one hand, *IL6*, *CXCL8*, *SOD1*, and *SSBP1* were significantly and negatively correlated with *ESR1* expression; instead, *SIRT3* was significantly and positively correlated. On the other hand, *IL6R*, *CXCR8*, *GPX1*, *SLC25A14*, *NRF1*, *PPARGC1A*, *SIRT1*, and *OMA1* were significantly and positively correlated with *ESR2* expression.

## 4. Discussion

In this study, the effects of obesity related-inflammation on mitochondrial functionality in breast cancer cell lines and breast tumors, focusing on estrogen receptors ratio, were analyzed. Moreover, invasiveness in this situation was also analyzed. We demonstrated that ERβ, in an inflammatory and obesity condition, maintains mitochondrial functionality and avoids invasiveness in breast cancer cell lines. Moreover, we found a strong correlation between interleukin-6 receptor gene expression and inflammation, mitochondrial functionality, and oxidative stress markers, as well as with estrogen receptor beta, in breast cancer human samples in different BMI situations. 

Obesity stimulates the adipose tissues to release inflammatory mediators such as tumor necrosis factor α and interleukin 6, predisposing them to a proinflammatory state and oxidative stress [14,39]. These signals also stimulate the release of inflammatory mediators by breast cancer cells, creating an autocrine feedback loop [40]. To start the study, we confirmed the effects of treatment on inflammatory genes expression. ELIT treatment (17β-estradiol (10 nM), Leptin (100 ng/mL), IL6 (50 ng/mL), and TNFα (10 ng/mL)), simulates circulating hormonal conditions in a postmenopausal obese woman and inducing a high increase in proinflammatory expression genes. Furthermore, it is worth noting that, in breast tumors in different BMI situations, inflammatory genes expression was positively correlated with interleukin-6 receptor gene expression. These results are in concordance with other studies where proinflammatory markers are studied in postmenopausal breast cancer patients [32].

Previous studies in our group and other research groups have shown the independent effects of 17β-estradiol or leptin on oxidative stress and mitochondrial biogenesis and dynamics [9,41,42,43,44], but never in an associated-inflammation situation. It is well known that ERα is the predominant estrogen receptor found in the MCF7 cell line, and it responds to estrogens by increasing proliferation, while, if ERβ is overexpressed in these cells, the proliferative effect of estrogens is inhibited [9]. Therefore, the response to estrogens in breast cancer not only depends on the concentration of estrogens in the cellular environment but also depends on the ERα/ERβ ratio presented by cells [9,43]. 

In this study, MCF7 and T47D cell lines have been treated with an ELIT inflammatory cocktail with the aim of generates an inflammation situation (IL6 and TNFα) in the presence of Leptin and 17β-estradiol levels, simulating the physiological condition of postmenopausal obese women. In this way, an increase in reactive oxygen species has been observed in both cell lines; however, in the T47D cell line, a lower increase in the production of hydrogen peroxide and levels of superoxide anion was observed. These data make more sense when studying the oxidative damage present in both cell lines, where a significant increase was only observed in the MCF7 cell line. On the other hand, it seems that the T47D treated cell line does not present differences compared to the control group, despite presenting higher levels of H_2_O_2_ and superoxide anion, as similarly described by some authors [41,45,46].

Oxidative stress is generated in breast cancer, and in many other pathologies, in two main ways. The first one, as observed in the high ERα/ERβ ratio MCF7 cell line after ELIt treatment, is a decrease in the expression of the antioxidant enzymes. It was observed that all antioxidant enzymes had decreased their gene or protein expression in the MCF7 cell line, except the SOD2 enzyme that had increased expression in both MCF7 and T47D cell lines. It has been described that SOD2 plays a role as a free radical detector, increasing gene and/or protein expression in order to alleviate oxidative damage [47]. Moreover, *NFE2L2*, a transcription factor that controls mainly antioxidant enzymes expression, and glutathione reductase, which recycles glutathione, was also increased, avoiding a high increase in free radical levels in low ERα/ERβ ratio T47D cell line. In addition, it should be noted that those tumors with a high correlation between *IL6R* and *ESR2* gene expression presented an increase in antioxidant enzymes, thus could palliate levels of free radicals, which can ultimately diminish oxidative damage.

The other pathway that increases oxidative stress is the poor maintenance of a functional mitochondrial pool [48]. To achieve good maintenance, two highly coordinated processes, such as mitochondrial biogenesis and dynamics, are very important [49,50]. In the MCF7 cell line, where ERα predominates, the protein levels of the OXPHOS system are decreased after ELIT treatment, as well as the amount of inner mitochondrial membrane (cardiolipin levels), required for the activity of complexes I, III, and IV, and plays an important role in mitochondrial biogenesis [51,52]. Likewise, both mitochondrial biogenesis and mitochondrial dynamics are diminished by ELIT treatment in the MCF7 cell line. Therefore, neither mitochondria are generated, nor are those that do not function properly eliminated; thus, the production of free radicals and, consequently, the oxidative damage is increased. Mitochondrial dysfunction has been postulated as one of the hallmarks of cancer, increasing free radicals and decrease energetic efficiency [19]. If this situation is accompanied by low levels of antioxidant enzymes, oxidative damage will be higher, as observed in the MCF7 cell line. 

However, the T47D cell line, with high levels of ERβ, had mitochondrial dynamics elevated, maintaining a more functional pool of mitochondria. In the case of mitochondrial biogenesis, it should be noted that there are two genes that presented a very significant elevation, estrogen-related receptor alpha (*ESRRA*) and Twinkle (*TWNK*). ESRRA belongs to a superfamily of nuclear receptors independent of estrogen activation. Its expression is induced and activated by PGC1α, and the two factors together are capable of binding to response elements and promoting the initiation and elongation of the transcription of metabolic and mitochondrial target genes [53]. Twinkle is the mitochondrial helicase encoded by nuclear DNA that acts during mtDNA replication, and its overexpression has been shown to be related to increased mitochondrial biogenesis [54]. In fact, ESRRA should be considered as a possible factor responsible for the difference in the rate of mitochondrial biogenesis between both MCF7 and T47D cell lines in an obese-related inflammation situation. As shown in the results, the increased expression of the nuclear receptor ESRRA in the T47D cell line could be responsible for the maintenance of the mitochondrial pool in the cell. Furthermore, it has been observed that the STAT3 signaling pathway, activated by leptin and IL6, upregulates the expression of ESRRA [55]. 

Taking into account that patients with tumors with low ERα/ERβ ratios have a worse response to chemical agents oxidative damage inductors [56], we observed that breast tumors with a high correlation between IL6R and ESR2 in obese patients had genes related to mitochondrial biogenesis, and dynamics increased gene expression, which could be an attempt to increase the number of mitochondria due to the inflammatory state and oxidative stress generated over a long period of time, such happens in other situations like aging [57,58], but maintaining a functional mitochondrial pool. In addition, it is worthy to note that both interleukin 6 and 8 gene expression correlated negatively with estrogen receptor alpha gene expression, whereas both interleukin 6 and 8 receptors correlated positively with estrogen receptor beta gene expression. These results remark the importance of more studies are needed in order to better understand estrogen receptors subtypes’ role in breast cancer. 

In addition to mitochondrial biogenesis and dynamics, our study reveals an oxygen consumption rate decreased in the MCF7 cell line that supports all the results commented above. It is worth noting that ELIT treatment decreases basal respiration, maximal respiratory capacity, ATP-linked respiration as much as proton leak in the MCF7 cell line. Proton leak decrease is in concordance with low uncoupling proteins mRNA expression levels found in the MCF7 cell line after ELIT treatment. UCPs, which promote proton leak across the inner mitochondrial membrane, have emerged as essential regulators of mitochondrial membrane potential, respiratory activity, and ROS generation [59]. As seen in our results, the mitochondrial network was modified in the MCF7 cell line after treatment. Mitochondria appear more fragmented and circular than fused in the ELIT-treated MCF7 cell line but not in the T47D cell line. In fact, it seems that estrogen receptor beta could maintain mitochondrial network as well as oxygen consumption rate. Moreover, the T47D cell line showed an increase in the reserve capacity of mitochondria, a fact that supports good mitochondrial pool maintenance [48,60,61]. 

Oxidative stress, inflammation, and poor mitochondrial network observed in MCF7 cell lines after ELIT treatment leads to a worse situation, and some authors have described a relationship between this situation and metastasis [62]. Our results are in concordance with this idea due to increased motility and modification of invasiveness markers presented in the MCF7 cell line but not in the T47D cell line after treatment. In fact, the T47D cell line, with a better mitochondrial profile, including biogenesis, dynamics, and mitochondrial network, showed high levels of Cadherin-E that could simulate an epithelial-like phenotype, as suggested by some authors [63,64].

As mentioned above, the MCF7 cell line has a higher ERα/ERβ ratio, and the T47D cell line has a lower ratio. ELIT-treatment decreases estrogen receptor alpha mRNA expression in both MCF7 and T47D cell lines, as shown in results. However, estrogen receptor beta mRNA expression only decreases in the MCF7 cell line but not in the T47D cell line. This fact supports that all the results showed in our study could be through estrogen receptor beta T47D cell lines maintenance, giving to this receptor subtype the protective role that previously some studies have been described [9,39]. 

## 5. Conclusions

The presence of estrogen receptor beta allows maintaining a more functional mitochondrial pool, with active mitochondrial biogenesis and dynamics, which means less production of reactive oxygen species and better mitochondrial metabolism in an obesity-related inflammation condition. In addition, antioxidant enzymes are active, preventing oxidative damage and, at least in part, invasiveness.

This study could be important to remark the importance of estrogen receptor beta and mitochondria as an important organelle in the development and prognosis of breast cancer in obese patients. Likewise, more studies are necessary in order to clarify the estrogen receptor beta mechanism in breast cancer and establish it as a clinical biomarker, as is the estrogen receptor alpha.

## Figures and Tables

**Table 1:** Primers and conditions used for RT-qPCR.

Gene Accession Number	Forward Primer (5′-3′)	An. T° (°C)
	Reverse Primer (5′-3′)	
*ESR2* *NM_001437.3*	TAG TGG TCC ATC GCC AGT TAT	60
	GGG AGC CAC ACT TCA CCA T	
*NFE2L2* *NM_006164.5*	GCG ACG GAA AGA AGT ATG AGC	60
	GTT GGC AGA TCC ACT GGT TT	
*CAT* *NM_001752.4*	CAT CGC CAC ATG AAT GGA TA	61
	CCA ACT GGG ATG AGA GGG TA	
*GSR* *NM_000637.5*	TCA CGC AGT TAC CAA AAG GA	64
	CAC ACC CAA GTC CCC TGC AT	
*SOD1* *NM_000454.5*	TCA GGA GAC CAT TGC ATC ATT	64
	CGC TTT CCT GTC TTT GTA CTT TCT TC	
*SOD2* *NM_000636.4*	CGT GCT CCC ACA CAT CAA TC	64
	TGA ACG TCA CCG AGG AGA AG	
*UCP2* *NM_001381943.1*	GGT GGT CGG AGA TAC CAA	60
	CTC GGG CAA TGG TCT TGT	
*TNF* *NM_000594.4*	AAG CCT GTA GCC CAT GTT GT	58
	GGA CCT GGG AGT AGA TGA GGT	
*PTGS2* *NM_000963.4*	CCC TTC TGC CTG ACA CCT TT	60
	TTC TGT ACT GCG GGT GGA AC	
*IL6* *NM_000600.5*	CAG GGG TGG TTA TTG CAT CT	60
	AGG AGA CTT GCC TGG TGA AA	
*CXCL8* *NM_000584.4*	GGC ACA AAC TTT CAG AGA CAG CAG	66
	GTT TCT TCC TGG CTC TTG TCC TAG	
*IL6R* *NM_000565.4*	TGG GAG GTG GAG AAG AGA GA	60
	AGG ACC TCA GGT GAG AAG CA	
*CXCR8* *NM_000634.3*	AGT TCT TGG CAC GTC ATC GT	58
	CCC CTG AAG ACA CCA GTT CC	
*TGFB* *NM_000660.7*	TCC TGG CGA TAC CTC AGC AA	60
	CGG TAG TGA ACC CGT TGA TG	
*NRF1* *NM_005011.5*	CCA CGT TAC AGG GAG GTG AG	60
	TGT AGC TCC CTG CTG CAT CT	
*SSBP1* *NM_001256510.1*	TGT GAA AAA GGG GTC TCG AA	60
	TGG CCA AAG AAG AAT CAT CC	
*PPARGC1A* *NM_001330751.2*	TCA GTC CTC ACT GGT GGA CA	60
	TGC TTC GTC GTC AAA AAC AG	
*TFAM* *NM_003201.3*	GTG GTT TTC ATC TGT CTT GGC	60
	ACT CCG CCC TAT AAG CAT CTT	
*TWNK* *NM_021830.5*	GGG AGG AGG TGC TAG GAG AA	61
	TTC CTG GCT TGC TTT GGC T	
*MFN1* *NM_033540.3*	TTC GAT CAA GTT CCG GAT TC	51
	TTG GAG CGG AGA CTT AGC AT	
*MFN2* *NM_014874.4*	GCA GAA CTT TGT CCC AGA GC	56
	AGA GGC ATC AGT GAG GTG CT	
*OPA1* *NM_015560.3*	ACA ATG TCA GGC ACA ATC CA	51
	GGC CAG CAA GAT TAG CTA CG	
*OMA1* *NM_145243.5*	TTG GAT TGC TCT TTG TGG TG	51
	GGT ATC GGG CAT CTT TCT CA	
*DNM1L* *NM_012062.5*	GTT CAC GGC ATG ACC TTT TT	51
	AAG AAC CAA CCA CAG GCA AC	
*FIS1* *NM_016068.3*	GCT GAA GGA CGA ATC TCA	55
	CTT GCT GTG TCC AAG TCC AA	
*SIRT1* *NM_012238.5*	GCA GAT TAG TAG GCG GCT TG	60
	TCT GGC ATG TCC CAC TAT CA	
*SIRT3* *NM_012239.6*	CGG CTC TAC ACG CAG AAC ATC	56
	CAG AGG CTC CCC AAA GAA CAC	
*GAPDH* *NM_002046.7*	CCA CTC CTC CAC CTT TGA CG	60
	CTG GTG GTC CAG GGG TCT TA	
*18S* *NR_146119.1*	GGACACGGACAGGATTGACA	60
	ACCCACGGAATCGAGAAAGA	
*STAT3* *NM_139276.3*	CTG GCC TTT GGT GTT GAA AT	61
	AAG GCA CCC ACA GAA ACA AC	
*SLC25A14* *NM_001282195.2*	CAA GCC GTT GGT CTC CTA AG	60
	CGT TTT CAA TGT CAC CCA TC	
*CDH1* *NM_004360.5*	GTCACTGACACCAACGATAATCCT	60
	TTTCAGTGTGGTGATTACGACGTTA	
*ESRRA* *NM_004451.5*	TCG CTC CTC CTC TCA TCA TT	52
	TGG CCA AAC CCA AAA ATA AA	
*PPARG* *NM_138712.5*	GAG CCC AAG TTT GAG TTT GC	61
	CTG TGA GGA CTC AGG GTG GT	
*ESR1* *NM_000125.4*	AAT TCA GAT AAT CGA CGC CAG	61
	GTG TTT CAA CAT TCT CCC TCC TG	
*MMP9* *NM_004994.3*	CGC AGA CAT CGT CAT CCA GT	60
	AAA CCG AGT TGG AAC CAC GA	
*GPER1* *NM_001505.3*	CAT CAT CGG CCT GTG CTA CT GAT GAA GAC CTT CTC CGG CA	60
*GPX1* *NM_000581.4*	GCG GCG GCC CAG TCG GTG TA	61
	GAG CTT GGG GTC GGT CAT AA	

**Table 2:** Oxidative stress in MCF7 and T47D breast cancer cell lines.

	MCF7		T47D	
	Control	ELIT	Control	ELIT
Superoxide anion levels (%)	100 ± 13	1177 ± 116 *	100 ± 16	526 ± 35 *
H_2_O_2_ production (%)	100 ± 1	200 ± 8 *	100 ± 2	134 ± 3 *
Cardiolipin content (%)	100 ± 2	82.7 ± 1.1 *	100 ± 1	101 ± 1
Oxidative damage (%)	100 ± 5	145 ± 8 *	100 ± 7	92.3 ± 11.7

Data represent the means ± SEM (*n* = 6). Values of control (DMSO-treated) cells were set at 100 in each cell line. * Significant difference between ELIT-treated and control cells (Student’s test; *p* < 0.05).

**Table 3:** Antioxidant enzymes protein levels in MCF7 and T47D breast cancer cell lines.

	MCF7		T47D	
	Control	ELIT	Control	ELIT
SOD1 (%)	100 ± 12	89 ± 14	100 ± 25	156 ± 28 *
SOD2 (%)	100 ± 11	1631 ± 552 *	100 ± 15	3691 ± 350 *
CAT (%)	100 ± 6	70 ± 8 *	100 ± 14	88 ± 10
GSR (%)	100 ± 14	49 ± 6 *	100 ± 9	117 ± 8

Data represent the means ± SEM (*n* = 6). Values of control (DMSO-treated) cells were set at 100 in each cell line. * Significant difference between ELIT-treated and control cells (Student’s test; *p* < 0.05).

**Table 4:** Mitochondrial-related protein levels in MCF7 and T47D breast cancer cell lines.

	MCF7		T47D	
	Control	ELIT	Control	ELIT
PPARGC1A (%)	100 ± 11	142 ± 2 *	100 ± 13	91 ± 16
Complex I (NDUFB8) (%)	100 ± 7	36 ± 2 *	100 ± 16	80 ± 21
Complex II (SDHB) (%)	100 ± 17	39 ± 4 *	100 ± 18	84 ± 10
Complex III (UQCRC2) (%)	100 ± 40	136 ± 24	100 ± 5	101 ± 8
Complex IV (COX II) (%)	100 ± 10	26 ± 4 *	100 ± 18	65 ± 13
(COX IV) (%)	100 ± 5	54 ± 6 *	100 ± 3	86 ± 13
Complex V (ATP5A) (%)	100 ± 9	56 ± 10 *	100 ± 14	117 ± 4

Data represent the means ± SEM (*n* = 6). Values of control (DMSO-treated) cells were set at 100 in each cell line. * Significant difference between ELIT-treated and control cells (Student’s test; *p* < 0.05).

**Table 5:** Correlation values of IL6R gene expression in breast tumors according to obesity status.

		nw	ow	o			nw	ow	o			nw	ow	o
IL6R	Pearson Correlation	1	1	1	CAT	Pearson Correlation	−0.205	0.421	0.164	SSBP1	Pearson Correlation	0.306	−0.218	0.229
	Sig.					Sig.	0.285	0.173	0.272		Sig.	0.195	0.319	0.197
ESR1	Pearson Correlation	−0.006	0.105	−0.337	GPX1	Pearson Correlation	0.358	0.406	0.704 **	NRF1	Pearson Correlation	0.870 **	0.878 **	0.936 **
	Sig.	0.494	0.412	0.101		Sig.	0.155	0.183	0.001		Sig.	0.001	0.005	0
ESR2	Pearson Correlation	0.396	0.703	0.605 *	GSR	Pearson Correlation	0.454	0.286	−0.009	PPARGC1A	Pearson Correlation	0.656 *	0.807 *	0.830 **
	Sig.	0.19	0.059	0.024		Sig.	0.094	0.267	0.486		Sig.	0.02	0.014	0
CXCR8	Pearson Correlation	0.791 **	0.840 **	0.957 **	SOD1	Pearson Correlation	0.438	0.062	0.434 *	SIRT1	Pearson Correlation	0.228	0.261	0.604 **
	Sig.	0.003	0.009	0		Sig.	0.103	0.448	0.046		Sig.	0.263	0.286	0.007
IL6R	Pearson Correlation	0.421	0.113	0.304	SOD2	Pearson Correlation	0.289	−0.459	−0.106	TFAM	Pearson Correlation	0.279	−0.037	0.387
	Sig.	0.113	0.405	0.126		Sig.	0.209	0.15	0.348		Sig.	0.234	0.468	0.069
CXCL8	Pearson Correlation	0.732 **	0.662	0.911 **	SIRT3	Pearson Correlation	0.428	−0.11	0.3	FIS1	Pearson Correlation	0.609 *	0.326	0.883 **
	Sig.	0.008	0.053	0		Sig.	0.125	0.407	0.129		Sig.	0.041	0.237	0
TGFB	Pearson Correlation	0.296	−0.552	0.272	NFE2L2	Pearson Correlation	0.680 *	0.753 *	0.812 **	OMA1	Pearson Correlation	0.379	0.472	0.541 *
	Sig.	0.203	0.1	0.154		Sig.	0.015	0.025	0		Sig.	0.157	0.172	0.015
TNF	Pearson Correlation	0.604 *	0.684 *	0.806**	UCP2	Pearson Correlation	0.256	−0.266	0.332	OPA1	Pearson Correlation	−0.39	−0.45	−0.032
	Sig.	0.032	0.045	0		Sig.	0.238	0.282	0.104		Sig.	0.17	0.224	0.456
PTGS2	Pearson Correlation	0.07	−0.134	0.639 **	SLC25A14	Pearson Correlation	0.874 **	0.901 **	0.940 **	* The correlation is significant at *p* < 0.05				
	Sig.	0.435	0.415	0.007		Sig.	0	0.003	0	** The correlation is significant at *p* < 0.01				

Data represent Pearson correlations and significance (unilateral) in normal weight (*n* = 9). overweight (*n* = 8) and obesity (*n* = 16) groups. * (*p* < 0.05) ** (*p* < 0.01) significant difference.

**Table 6:** Correlation values of estrogen receptors subtype alpha and beta gene expression in breast tumors. (**a**) *ESR1* correlation. (**b**) *ESR2* correlation.

(a)								
ESR1	Pearson Correlation	1.000	CAT	Pearson Correlation	0.089	SSBP1	Pearson Correlation	−0.461 **
	Sig.			Sig.	0.624		Sig.	0.007
IL6R	Pearson Correlation	−0.219	GPX1	Pearson Correlation	0.218	NRF1	Pearson Correlation	−0.154
	Sig.	0.221		Sig.	0.223		Sig.	0.393
ESR2	Pearson Correlation	0.010	GSR	Pearson Correlation	0.139	PPARGC1A	Pearson Correlation	−0.314
	Sig.	0.962		Sig.	0.440		Sig.	0.075
CXCR8	Pearson Correlation	−0.230	SOD1	Pearson Correlation	−0.391 *	SIRT1	Pearson Correlation	−0.012
	Sig.	0.205		Sig.	0.024		Sig.	0.945
IL6	Pearson Correlation	−0.364 *	SOD2	Pearson Correlation	0.177	TFAM	Pearson Correlation	−0.003
	Sig.	0.037		Sig.	0.323		Sig.	0.989
CXCL8	Pearson Correlation	−0.422 *	SIRT3	Pearson Correlation	0.571 **	FIS1	Pearson Correlation	−0.192
	Sig.	0.014		Sig.	0.001		Sig.	0.292
TGFB	Pearson Correlation	0.221	NFE2L2	Pearson Correlation	−0.122	OMA1	Pearson Correlation	0.110
	Sig.	0.216		Sig.	0.500		Sig.	0.555
TNF	Pearson Correlation	0.041	UCP2	Pearson Correlation	0.153	OPA1	Pearson Correlation	0.125
	Sig.	0.820		Sig.	0.396		Sig.	0.525
PTGS2	Pearson Correlation	−0.200	SLC25A14	Pearson Correlation	−0.163	* The correlation is significant at *p* < 0.05		
	Sig.	0.317		Sig.	0.364	** The correlation is significant at *p* < 0.01		
**(b)**								
ESR2	Pearson Correlation	1.000	CAT	Pearson Correlation	SSBP1		Pearson Correlation	0.260
	Sig.			Sig.			Sig.	0.220
IL6R	Pearson Correlation	0.639 **	GPX1	Pearson Correlation	NRF1		Pearson Correlation	0.483 *
	Sig.	0.001		Sig.			Sig.	0.017
ESR1	Pearson Correlation	0.010	GSR	Pearson Correlation	PPARGC1A		Pearson Correlation	0.409 *
	Sig.	0.962		Sig.			Sig.	0.047
CXCR8	Pearson Correlation	0.473 *	SOD1	Pearson Correlation	SIRT1		Pearson Correlation	0.560 **
	Sig.	0.023		Sig.			Sig.	0.004
IL6	Pearson Correlation	−0.060	SOD2	Pearson Correlation	TFAM		Pearson Correlation	0.326
	Sig.	0.781		Sig.			Sig.	0.120
CXCL8	Pearson Correlation	0.374	SIRT3	Pearson Correlation	FIS1		Pearson Correlation	0.294
	Sig.	0.071		Sig.			Sig.	0.163
TGFB	Pearson Correlation	0.169	NFE2L2	Pearson Correlation	OMA1		Pearson Correlation	0.446 *
	Sig.	0.430		Sig.			Sig.	0.029
TNF	Pearson Correlation	0.326	UCP2	Pearson Correlation	OPA1		Pearson Correlation	0.256
	Sig.	0.120		Sig.			Sig.	0.262
PTGS2	Pearson Correlation	−0.155	SLC25A14	Pearson Correlation	* The correlation is significant at *p* < 0.05			
	Sig.	0.502		Sig.	** The correlation is significant at *p* < 0.01			

## Data Availability

Data is contained within the article and Supplementary Materials.

## Supplementary Materials

The following are available online at https://www.mdpi.com/article/10.3390/antiox10091371/s1, Figure S1: Representative bands and values of Inmunoblotting against GAPDH (approximately 37 kDa). Order of wells and Relative intensity values are above the blot image, control situation of each cell line set as 100; Figure S2: Representative bands and values of Inmunoblotting against 4HNE. Order of wells and Relative intensity values are above the blot image, control situation of each cell line set as 100; Figure S3: Representative bands and values of Inmunoblotting against SOD1 (Approximately 16 kDa). Order of wells and Relative intensity values are above the blot image, control situation of each cell line set as 100; Figure S4: Representative bands and values of Inmunoblotting against SOD2 (Approximately 25 kDa). Order of wells and Relative intensity values are above the blot image, control situation of each cell line set as 100; Figure S5: Representative bands and values of Inmunoblotting against GRd (Approximately 56 kDa). Order of wells and Relative intensity values are above the blot image, control situation of each cell line set as 100; Figure S6: Representative bands and values of Inmunoblotting against CAT (Approximately 60 kDa). Order of wells and Relative intensity values are above the blot image, control situation of each cell line set as 100; Figure S7: Representative bands and values of Inmunoblotting against OXPHOS (Cocktail antibody was used). Order of wells are above the blot image and Relative intensity values in table, control situation of each cell line set as 100; Figure S8: Representative bands and values of Inmunoblotting against COXIV; Figure S9: Illustrations of Wound Healing in both MCF7 and T47D cell lines after 0, 12 and 24 h of ELIT treatment.
