# Supplementary material for: Estrogen Receptor Beta (ERβ) Maintains Mitochondrial Network Regulating Invasiveness in an Obesity-Related Inflammation Condition in Breast Cancer
Source: Antioxidants (Basel). 2021 Aug 28;10(9):1371. doi: 10.3390/antiox10091371 (PMC8466315; doi:10.3390/antiox10091371)
Supplement: Supplementary file 1 [file antioxidants-10-01371-s001.zip › antioxidants-1342305-SI.pdf]

Supplementary Materials

Estrogen receptor beta (ERβ) maintains mitochondrial network regulating invasiveness in an obesity-related inflammation condition in breast cancer.

Toni Martinez-Bernabe[1,2‡], Jorge Sastre-Serra[1,2,3‡], Nicolae Ciobu[1], Jordi Oliver[1,2,3], Daniel Gabriel Pons[1,2,*] and Pilar Roca[1,2,3]

Order: MCF7C/MCF7ELIT/MW/T47DC/T47DELIT/MCF7C/MCF7ELIT/T47DC/T47DELIT
Rel. Int.: 36 / 127 / X / 117 / 108 / 106 / 120 / 94 / 92

IB: Anti-GAPDH

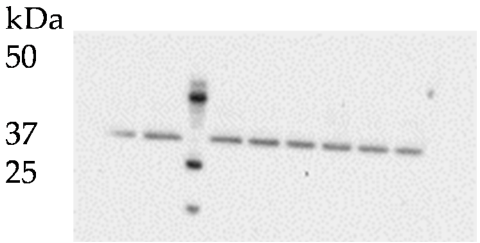

Figure S1. Representative bands and values of Inmunoblotting against GAPDH (approximately 37 kDa). Order of wells and Relative intensity values are above the blot image, control situation of each cell line set as 100.

Order: MCF7C/MCF7ELIT/ MCF7C/MCF7ELIT /T47DC/T47DELIT/T47DC/T47DELIT/MW
Rel. Int.: 104 / 152 / 100 / 133 / 109 / 115 / 108 / 108 / X

IB: Anti-4HNE

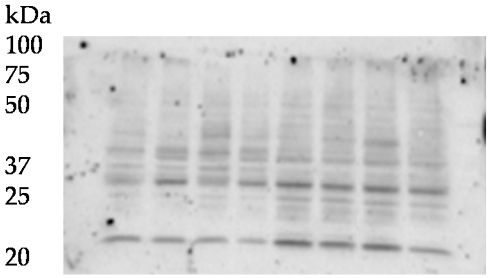

Figure S2. Representative bands and values of Inmunoblotting against 4HNE. Order of wells and Relative intensity values are above the blot image, control situation of each cell line set as 100.

Order: T47DC/MW/T47DELIT/T47DC/T47DELIT/MCF7C/MCF7ELIT/MCF7C/ X
Rel. Int.: 103 / 115 / 124 / 175 / 97 / 103 / 126 / 58 / X

IB: Anti-SOD1

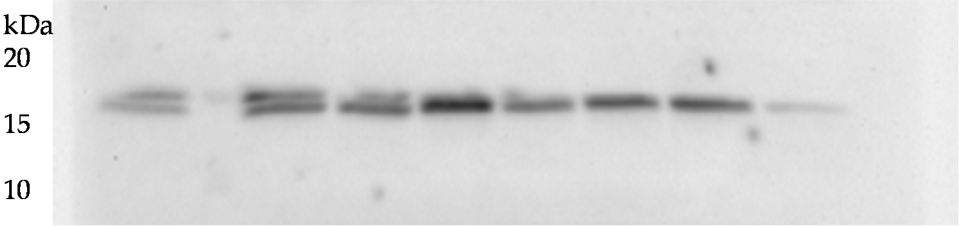

Figure S3. Representative bands and values of Inmunoblotting against SOD1 (Approximately 16 kDa). Order of wells and Relative intensity values are above the blot image, control situation of each cell line set as 100.

Order: MCF7C/MCF7ELIT/MW/T47DC/T47DELIT/MCF7C/MCF7ELIT/T47DC/T47DELIT
Rel. Int.: 110 / 1213 / X / 108 / 4648 / 115 / 1189 / 91 / 3995

IB: Anti-SOD2

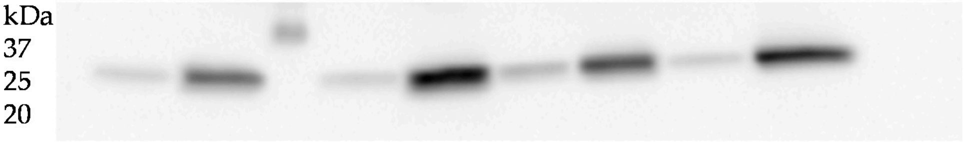

Figure S4. Representative bands and values of Inmunoblotting against SOD2 (Approximately 25 kDa). Order of wells and Relative intensity values are above the blot image, control situation of each cell line set as 100.

Order: MCF7C/MCF7ELIT/MW/T47DC/T47DELIT/MCF7C/MCF7ELIT/T47DC/T47DELIT
Rel. Int.: 111 / 45 / X / 114 / 111 / 91 / 49 / 98 / 110

IB: Anti-GRd

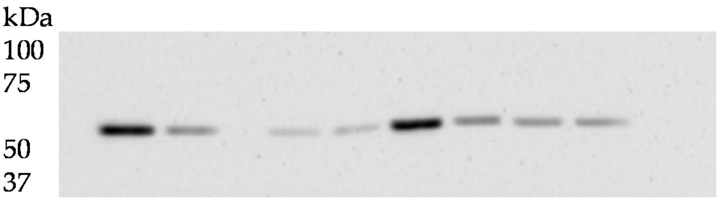

Figure S5. Representative bands and values of Inmunoblotting against GRd (Approximately 56 kDa). Order of wells and Relative intensity values are above the blot image, control situation of each cell line set as 100.

Order: MW/MCF7C/MCF7ELIT/T47DC/T47DELIT/MCF7C/MCF7ELIT/T47DC/T47DELIT
Rel. Int.: X / 97 / 50 / 106 / 60 / 113 / 99 / 105 / 68

IB: Anti-CAT

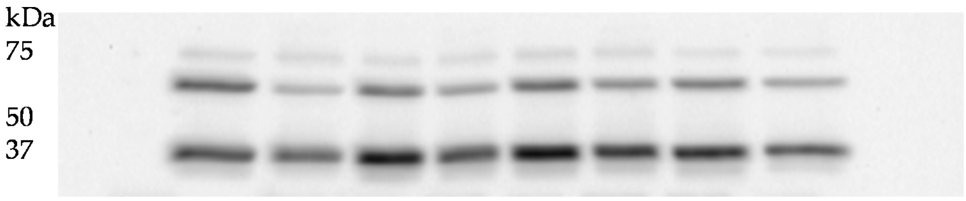

Figure S6. Representative bands and values of Inmunoblotting against CAT (Approximately 60 kDa). Order of wells and Relative intensity values are above the blot image, control situation of each cell line set as 100.

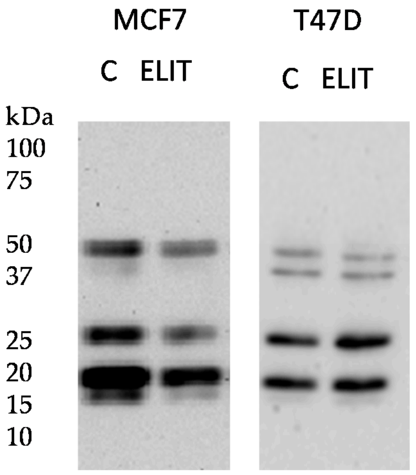

MCF7		T47D	
C	ELIT	C	ELIT
104	64	102	109
100	88	109	111
122	44	98	111
94	33	94	108
106	37	101	104

Figure S7. Representative bands and values of Inmunoblotting against OXPHOS (Cocktail antibody was used). Order of wells are above the blot image and Relative intensity values in table, control situation of each cell line set as 100.

Order: 1 - MW / MCF7C / MCF7ELIT / MCF7C / MCF7ELIT / MCF7C / MCF7ELIT
2 – T47DC /MK / T47DELIT / T47DC / T47DELIT / T47DC / x / T47DC/ T47DELIT

IB: Anti - COXIV

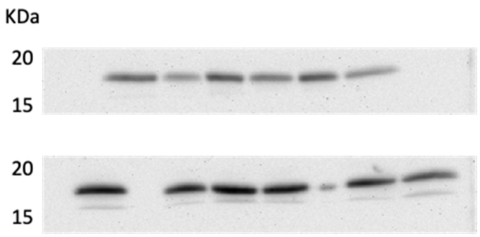

Figure S8. Representative bands and values of Inmunoblotting against COXIV.

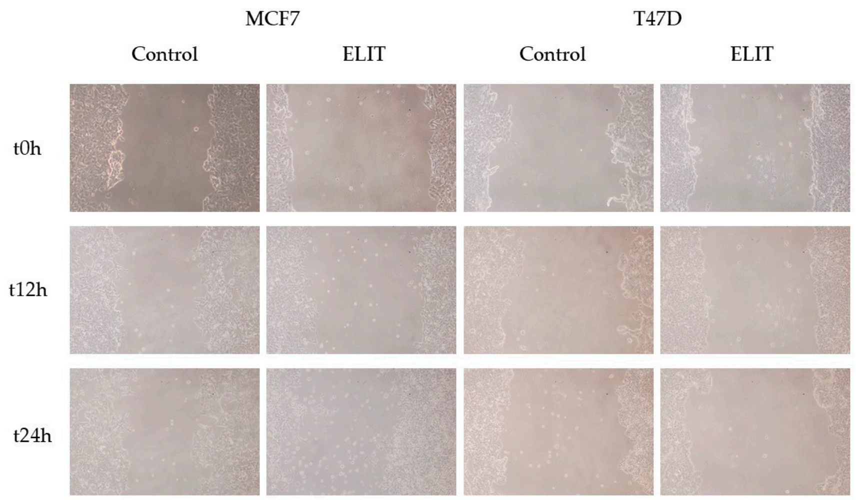

Figure S9. Illustrations of Wound Healing in both MCF7 and T47D cell lines after 0, 12 and 24 hours of ELIT treatment.

© 2021 by the authors. Submitted for possible open access publication under the terms and conditions of the Creative Commons Attribution (CC BY) license (http://creativecommons.org/licenses/by/4.0/).
